# Factors affecting milk cortisol in mid lactating dairy cows

**DOI:** 10.1186/s12917-015-0572-9

**Published:** 2015-10-12

**Authors:** Sandy Sgorlon, Marta Fanzago, Denis Guiatti, Gianfranco Gabai, Giuseppe Stradaioli, Bruno Stefanon

**Affiliations:** Dipartimento di Scienze Agrarie e Ambientali, Università di Udine, via delle Scienze 208, 33100 Udine, Italy; Dipartimento di Biomedicina Comparata ed alimentazione, Università di Padova, viale dell’Università 16, 35020 Legnaro, Italy

**Keywords:** Milk cortisol, Somatic cell count, Health, Dairy cow, Breed

## Abstract

**Background:**

Whether the measurement of cortisol in dairy cows can be used as a biomarker of adverse environmental or pathophysiological conditions is still under of scientific debate. In these situations, several systems mainly the hypothalamic-pituitary-adrenal axis, the autonomic nervous system, and the immune system are recruited to reestablish homeostasis. A first aim of the present study was to compare milk and blood cortisol concentrations and to consider its variability in milk in relation to farm, milk yield and days in milk. A second study investigates the effects of breed, class of somatic cell count (SCC) and farm on milk cortisol levels in a larger number of cows and farms, with the aim to validate the results obtained in the pilot study.

**Methods:**

For study 1, 135 cows were sampled from 2 Italian Simmental and 2 Italian Holstein commercial farms, whilst in the second study, 542 cows were sampled from 6 commercial farms of Italian Simmental and 499 cows from 4 commercial farms of Italian Holstein.

**Results:**

In study 1, the values of cortisol content in milk were significantly higher in Holstein than Simmental cows. Significant differences between farms were observed for milk and plasma cortisol concentrations. Cortisol content in milk was not correlated to plasma content in study 1 and the mean milk to plasma cortisol ratio was about 1:30. In study 2, for Holstein cows, significantly higher values of milk cortisol in comparison to Simmental cows was reported. A significant effect of class of SCC was observed, cows belonging to class 3 (SCC higher than 400.000/ml) showed the highest mean values of milk cortisol. The farm effect was significant also in the study 2, confirming the results obtained in the first study.

**Conclusions:**

Milk can be considered a preferential site of sampling in dairy cows to point out short term stimulation of the hypothalamic-pituitary-adrenal axis. Further studies are needed to investigate the physiological basis of the relationship between milk cortisol content and breed, milk yield and SCC to ascertain the relevance of milk cortisol to monitor the healthy status of mammary gland.

## Background

The increasing levels of milk production of high genetic merit cow has been associated with impairment of fertility, longevity and incidence of diseases [1, 2]. The relationship between genetic merit and health is not well determined, but a recent study of multiple immune functions in lactating cows suggested association among immune traits with health events and fitness of dairy cows [3, 4].

Under challenging situations, such as disease, negative energy balance and perturbation of environmental conditions, several systems mainly the hypothalamic-pituitary-adrenal (HPA) axis, the autonomic nervous system, and the immune system are recruited to reestablish homeostasis. Stimulation of HPA axis leads to the secretion of various hormones that regulates target genes [5, 6] and differentially affects the immune system and blood constituents with consequences that depend from the type of stimulus, the species, the sex, and the individual considered [7, 8].

Whether the measurement of cortisol in dairy cows can be used as a biomarker of adverse environmental or pathophysiological conditions, which can negatively affect productive performances and welfare, is still under of scientific debate. Bertulat et al. [9] have reported higher concentration of glucocorticoid metabolites in the feces of drying off cows with higher milk yield and Horst & Jorgensen [10] reported an increase of plasma cortisol in cows with milk fever, associated with immune suppression and increase risk of clinical mastitis and high somatic cell count in milk.

Plasma cortisol is affected by sampling technique and sudden environmental modifications, suffering from the pulsatile secretion of the biomarker. Cortisol and its metabolites can be measured in integuments and fluids, as hair, urine, feces, and milk [9, 11, 12], each site of sampling presenting advantages and limitations. However, for lactating cows, milk can be viewed as the sampling site of first choice, since it could be measured without manipulation of animals, hence it is completely compatible with animal welfare recommendations. Even though no information is available on the prediction of milk cortisol with infrared spectroscopy or other sensors, milk offers the advantage to monitor the concentration of this hormone in line or at the official milk recording. These sampling strategies can provide different information about individual animals within a herd or between herds, and need different independent validation of milk cortisol as stress biomarker. The present work focused on factors affecting milk cortisol concentrations when samples are collected at the official milk recording. In particular, a first aim was to compare cortisol concentrations in milk and blood of Holstein and Simmental lactating cows at a farm level to evaluate the mean values and their ranges in field conditions and compare the variability of the biomarker between these sites of sampling. A second study aimed to investigate the variability of milk cortisol between breeds, herds and somatic cell count (SCC) in commercial farms measured at the official milk recording. To limit the potential variations of milk cortisol due to metabolic stress and related diseases, samples were collected after the peak of lactation, when cows approach a null or positive energy balance.

## Methods

### Animals and diets

Animals were sampled from commercial dairy farms located in the North East part of the Po Valley, Italy, presenting homogeneous management and ration compositions. Farms were selected together with the local Farm and Breeder Association (Associazione Allevatori del Friuli Venezia Giulia, Codroipo, Italy; www.aafvg.it), which provided also information about individual milk records, reproductive parameters and managerial aspects. Farmers and farm veterinary practitioners gave an oral informed consent to the animal study, and all the data obtained from the laboratory analyses were delivered to the farmers in written form. All the farms involved in the present study adhere to a high standard of veterinary care based on best practice manual, under the supervision of the official veterinary service.

For the first study, 2 commercial farms with Italian Simmental (IS) cows (Farm A = FA and Farm B = FB) and 2 commercial farms of Italian Holstein (IH) cows (Farm C = FC and Farm D = FD) were selected. The herd size was 330 for FA, 283 for FB, 347 for FC and 427 for FD. Since the aim of this trail was to compare milk and blood cortisol concentrations and to consider the distribution of the values of milk cortisol, a subset of 20 % of lactating cows with 70 < DIM < 250 (DIM = days in milking) and free from clinical diseases were randomly sampled. For the second study, 6 commercial farms of IS (Farm 2 = F2, Farm 3 = F3, Farm 6 = F6, Farm 7 = F7, Farm 8 = F8 and Farm 9 = F9) and 4 commercial farms of IH (Farm 1 = F1, Farm 4 = F4, Farm 5 = F5 and Farm 10 = F10), with a herd size ranging from 157 to 654 cows, were selected.

The inclusion criteria considered for the cows was to be clinical healthy and with 50 < DIM < 270. Details of herd compositions and farm characteristics are reported in Tables 1 and 2.

**Table 1:** Composition of the herds and characteristics of the farms involved in the study 1

		Farm			
		FA	FB	FC	FD
Breed		IS	IS	IH	IH
Herd size	N	330	283	347	427
Dairy animals	N	180	139	185	231
First calving	N	63	29	55	79
Lactating cows	N	153	121	173	217
Cows > 70	N	122	105	159	195
Cows sampled	N	27	33	36	39
	%	46	43	35	34
DIM	Mean	126.7	141.4	151	145.8
	sd	33	33.8	33.1	28.5
Housing	Type	Free Stall	Free Stall	Free Stall	Free Stall
Bedding	Type	Concrete	Straw	Concrete	Concrete
Milking	Type	Parlour	Parlour	Parlour	Parlour

*IH* Italian Holstein, *IS* Italian Simmental, *N* number, *FA to FD* farm A to farm D, *sd* standard deviation

**Table 2:** Composition of the herds and characteristics of the farms involved in the study 2

		FARM									
		F1	F4	F5	F10	F2	F3	F6	F7	F8	F9
Breed		IH	IH	IH	IH	IS	IS	IS	IS	IS	IS
Herd size	N	654	390	442	456	270	341	320	538	157	201
Dairy animals	N	347	236	235	250	147	194	163	280	88	119
First calving	N	131	74	82	85	36	64	51	86	18	41
Lactating cows	N	313	208	195	227	123	153	137	225	74	96
Cows > 50 DIM	N	279	186	147	204	111	144	117	185	64	84
Cows sampled	N	184	128	75	112	88	95	92	126	63	78
Housing	Type	Free stall	Free stall	Free stall	Free stall	Free stall	Free stall	Free stall	Free stall	Free stall	Free stall
Bedding	Type	Concrete	Concrete	Concrete	Concrete	Straw	Straw	Concrete	Concrete	Concrete	Concrete
Milking	Type	Parlour	Parlour	Parlour	Parlour	Parlour	Parlour	Parlour	Parlour	Parlour	Parlour

*IH* Italian Holstein, *IS* Italian Simmental, *N* number, *F1 to F10* farm 1 to farm 10

All the lactating cows were housed in free stalls with cubicles and milking parlour and the management of the farms was similar. Only for F2 and F3 of study 2, cows were in straw bedding. Cows had free access to water and a *ad libitum* total mixed ration (TMR) based on corn silage and formulated to cover nutrient requirements [13] was offered twice a day, after the morning and the afternoon milking. The composition of the rations and the amounts offered were recorded from the register of the TMR mixed feeder, starting from 1 week before the day of sampling. Samples of TMR were collected the day of sampling from the manger and were analyzed to calculate nutritive values, to ensure that energy and protein requirements were satisfied. The day of sampling, individual milk yield was measured and the body condition score (BCS) of each cow was recorded by the same experienced observer on a scale from 1 (thin) to 5 (fat) with 0.25 point intervals [14].

### Sample collection

The day of official milk recording of the Breeder Association, 100 ml of milk samples were collected by the technician of the Farm and Breeder Association in the parlour from each cow at the morning milking. An aliquot of 50 ml of milk was transferred into a tube containing preservative and was used for protein, fat, lactose analyses and for SCC determination, as required by the protocol for the official recording. The remaining aliquot, approximatively 50 ml of milk was transferred to a tube without preservative, frozen within 2 h and stored at −20 °C for cortisol analyses. After milking and before the morning meal, when cows had *ad libitum* access to fresh water and spontaneously moved to cattle feed headlocks fence, blood was sampled from the coccygeal vein in 10 ml vacuum tubes with K3-EDTA (Venoject, Terumo Europe N.V., Leuven, Belgium). Blood was centrifuged within 1 h at 1500 x g for 10 min at 20 °C and plasma samples were stored at −20 °C for cortisol analyses. All experimental procedures and the care of the animals complied to the Italian legislation on animal care (DL n.116, 27/1/1992) and adhered to the internal rules of University of Udine. The approval for conducting this study was also granted by the veterinarian responsible of animal welfare of the Department of Agricultural and Environmental Science of the University of Udine.

### Cortisol assay

Skimmed milk was previously obtained by centrifugation (1,500 × g, 4 °C, 15 min). Cortisol was extracted two times from skim milk (0.2 mL) with 4.0 mL dichloromethane in a glass tube. The mixture was shaken at 250 × g for 15 min in a shaker, and the supernatant solution was transferred into a fresh glass tube. The extracted solution was evaporated by heating in a hot water bath (50 °C) for 2 h. After complete drying, 0.1 mL assay buffer (PBS, 0.1 % BSA, pH 7.4), 0.1 mL borate buffer (boric acid 1.55 g in 500 mL distilled H_2_O, 0.1 % BSA, pH 7.4) added with 0.01 g thimerosol (sodium ethylmercurithiosalicylate; Sigma-Aldrich) were put into the tube and mixed by shaker for 10 min [15]. Plasma samples (0.1 mL) were extracted with 8 mL diethyl ether. The ether fractions were transferred into fresh glass tubes and dried under nitrogen. The dry extracts were carefully dissolved in 0.2 ml assay buffer [16].

Skim milk (0.05 mL) and plasma (0.1 mL) extracts were assayed by a solid-phase microtitre RIA [16]. Briefly, a 96-well microtitre plate (Optiplate, Perkin-Elmer Life Science, Boston, MA, USA) was coated with anti-rabbit γ − globulin serum raised in a goat, by incubating overnight the antiserum diluted 1:1000 in 0.15 mM sodium acetate buffer, pH 9, at 4 °C. The plate was then washed twice with PBS 0.1 % BSA, pH 7.4 (RIA buffer) and incubated overnight at 4 °C with 0.2 mL of the anti-cortisol serum diluted 1:8000. The antiserum (Centro Medico Diagnostico Emilia, Bologna, Italy) was raised in the rabbit against cortisol-3 carboxymethyloxime–BSA and showed the following cross reactions: cortisol 100 %, prednisolone 44.3 %, 11-deoxycortisol 13.9 %, cortisone 4.9 %, corticosterone 3.5 %, progesterone <0.01 %.

The plate was carefully washed with RIA buffer, and standards (1.56–400 pg/well), quality control, unknown extracts and tracer (1,2,6,7–3H-cortisol, Perkin-Elmer Life Sciences, 30 pg/well, specific activity: 3700 GBq/mmol) were added (final volume: 0.2 mL). The plate was incubated overnight at 4 °C, the incubation mixture was decanted and wells washed with RIA buffer, added with 200 μl scintillation cocktail (Microscint 20, Perkin-Elmer Life Sciences) and counted on the beta-counter (Top-Count, Perkin-Elmer Life Sciences). All samples were assayed in duplicate. The sensitivity of the assay was defined as the dose of hormone at 90 % binding (B/B0) and was 3.125 pg/well. The intra-assay and inter-assay coefficients of variation in high and low cortisol pooled plasma samples were 5.9 % and 9.1 % and 13.5 % and 15.1 %, respectively.

### Data calculation and statistical analysis

All the data were stored in a spreadsheet using Microsoft Office Excel (2010, Microsoft Corp., Redmond, WA) and statistical analyses were performed with the SPSS package [17].

Cows were classified in 3 groups according to the somatic cell count measured at the time of sampling. Class 1 grouped the cows with a value of SCC lower than or equal to 200,000 cells/ml of milk (healthy), Class 3, grouped the cows with a value of SCC higher than or equal to 400,000 cells/ml of milk and Class 2 grouped the cows with intermediate value of SCC (200,000 < cell/ml of milk < 400,000). Before analysis of variance, normality of independent variables was tested by the Kolmogorov-Smirnov non parametric test. Cortisol concentrations in plasma for trial 1 and in milk for trials 1 and 2 were not normal distributed and a natural logarithm transformation was applied before statistical analysis.

The following univariate analysis of variance was used:

$$ {\mathrm{Y}}_{\mathrm{i}\mathrm{jk}\mathrm{z}} = \upmu + \mathrm{Clas}{\mathrm{s}}_{\mathrm{i}} + \mathrm{Bree}{\mathrm{d}}_{\mathrm{j}} + \mathrm{Far}{\mathrm{m}}_{\mathrm{k}} + \mathrm{a}*\mathrm{D}\mathrm{I}{\mathrm{M}}_{\mathrm{i}\mathrm{jk}} + \mathrm{b}*\mathrm{M}{\mathrm{Y}}_{\mathrm{i}\mathrm{jk}} + {\upvarepsilon}_{\mathrm{i}\mathrm{jk}\mathrm{z}} $$

Where:

Y_ijkz_:  = dependent variable
μ:  = general mean
Class_i_:  = Fixed effect for Class of SCC, with i from 1 to 3
Breed_j_:  = Fixed effect for Breed, with j from 1 to 2
Farm_k_:  = Random effect for the Farm, with k from 1 to 4 in trial 1 and 1 to 10 in trial 2
a:  = linear effect for days in milking (DIM)
b:  = linear effect of milk yield (MY)
ε_ijkz_:  = residual error

Least square difference test was applied to assess significant differences between means.

## Results

In the first study, 27 and 33 IS cows were sampled from farms FA and FB, and 36 and 39 IH cows were sampled from farms FC and FD, corresponding to 34–46 % of animals within 70 < DIM < 250 (Table 1). Cows were housed in a freestall barn with cubicles for FA, FC and FD and straw for FB. The ingredients of the rations for the farms were corn silage, ground corn, alfalfa hay, solvent extracted soybean meal and protein supplements, based on this latter feed. The chemical compositions of the diets did not vary largely between farms. The CP content ranged from 14.7 to 15.7 % of DM, the NDF from 31.9 to 34.4 % and the starch from 25.3 to 27.9 % on DM basis.

In the second study, the cows sampled for each farm ranged from 75 to 184 for IH and from 63 to 126 for IS, corresponding to 60.2 and 80.6 % of the lactating cows within 50 < DIM < 270 for IH and IS, respectively (Table 2). The differences in the percentage were due to the shorter lactation length of the IS cows compared to IH cows that averaged 300 and 345 days, respectively. The ingredients of the rations for the 10 farms were similar to that of the farms of study 1, including corn silage, ground corn, alfalfa hay, solvent extracted soybean meal and protein supplements, based on this latter feed. The chemical compositions of the diets varied, on DM basis, from 12.4 to 14.8 % of DM for CP, from 35.4 to 42.6 % for NDF and from 20.8 to 30.1 % for starch.

The mean DIM values of the sampled cows in study 1 varied from 126.7 days of FA to 151.0 days of FD, corresponding to the mid lactation phase. For the second study, the mean DIM values of cows sampled were lower than that of study 1 and ranged from 110.6 to 178.8, because the inclusion criteria considered all the healthy cows with 50 > DIM > 270 and also the first calving cows (Table 3).

**Table 3:** Mean values of body condition score (BCS), days in milking (DIM), milk yield, milk fat and protein percentages and somatic cell counts (SCC) of the lactating cows of the study 1 and study 2

Farm	Breed	N. Obs	BCS score	DIM days	Milk yield kg/day	Fat %	Protein %	SCC ln
Trial 1		134	2.89	142.3	33.6	3.80	3.26	
FA	IS	26	3.29^A^	126.7^Ns^	31.0^B^	3.57^Ns^	3.41^A^	3.54^Ns^
FB	IS	33	2.89^B^	141.4^Ns^	31.7^B^	4.03^Ns^	3.48^A^	4.84^Ns^
FC	IH	36	2.91^B^	151.0^Ns^	31.9^B^	3.90^Ns^	3.00^C^	4.89^Ns^
FD	IH	38	2.61^C^	145.8^Ns^	38.5^A^	3.68^Ns^	3.24^B^	4.94^Ns^
MSE			0.101	1022.220	36.996	0.543	0.061	2.132
F1	IH	180	2.40^D^	178.8^A^	42.9^A^	3.25^D^	3.11^E^	4.96^B^
F4	IH	128	2.37^DE^	177.1^A^	27.6^D^	3.63^BC^	3.09^E^	4.37^C^
F5	IH	75	2.23^E^	110.6^E^	33.7^C^	3.49^C^	3.13^E^	4.87^BC^
F10	IH	110	2.40^D^	178.6^A^	36.9^B^	4.14^A^	3.39^DC^	4.73^BC^
F2	IS	88	2.82^B^	166.3^AB^	27.8^D^	3.48^C^	3.53^B^	4.68^BC^
F3	IS	93	2.95^B^	167.5^AB^	26.2^DE^	3.94^AB^	3.31^D^	5.11^AB^
F6	IS	92	2.87^B^	131.8^DE^	25.4^E^	3.95^A^	3.46^BC^	3.78^D^
F7	IS	125	3.01^B^	160.1^AB^	26.9^DE^	3.74^AB^	3.68^A^	5.47^A^
F8	IS	61	2.58^C^	140.6^CD^	24.3^E^	3.89^AB^	3.37^DC^	4.40^C^
F9	IS	74	3.44^A^	150.8^BCD^	30.7^CD^	3.58^BC^	3.53^B^	3.72^D^
MSE			0.182	4049.791	61.182	0.419	0.087	1.898

*IH* Italian Holstein, *IS* Italian Simmental, *FA to FD* farm A to farm D, *F1 to F10* farm 1 to farm10

*MSE* Mean Square Error, *DIM* days in milking. For trial 1 ^A^, ^B^, ^C^ on the same column denote significant differences for *P* < 0.01; For trial 1 ^A^, ^B^, ^C^, ^D^, ^E^ on the same column denote significant differences for *P* < 0.01; ^Ns^ not significant

The mean values of BCS, DIM, milk yield, milk fat and protein percentages and SCC for the cows sampled in study 1 and study 2 are summarized in Table 3. The mean values of BCS, DIM, milk yield and protein percentage varied between farms (*P* < 0.01) in study 1, and for protein percentage IS showed higher values than IH cows. Significant differences between farms were calculated for all the variables in study 2 (*P* < 0.01) and IH cows showed mean BCS values lower than IS cows.

**Fig. 1:**
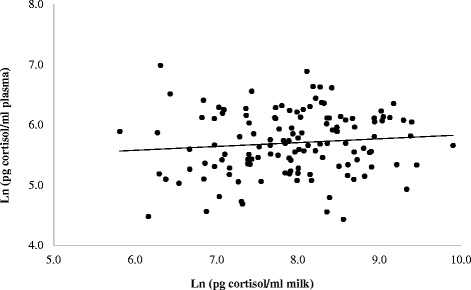
Correlation between milk and plasma cortisol (ln of pg/ml) concentrations (*r* = 0.109; not significant). The figure shows on the x-axis the concentration of cortisol in milk (ln of pg/ml) and on the y-axis the concentration of cortisol in plasma (ln of pg/ml) of 135 cows sampled from 2 Italian Simmental (IS) and 2 Italian Holstein (IH) commercial farms. The animals sampled were clinically healthy lactating cows with 70 < DIM < 250 (DIM = days in milking). Milk and plasma samples were collected at the morning milking before feed consumption

The effect of breed, class of SCC and farm on cortisol concentration in milk and plasma in study 1 is reported in Table 4. The values of cortisol content in milk were higher in IH than IS cows (*P* < 0.05), whereas plasma cortisol did not differ between breeds. Differences for milk and plasma cortisol concentrations between classes of SCC were not significant, while a significant effect was shown between farms (Table 4; *P* < 0.01). No significant effects (Table 4) of covariates DIM and milk yield were calculated. Cortisol content in milk was not correlated to plasma content (Fig. 1, r = 0.109, *P* > 0.05) in study 1 and the mean milk to plasma cortisol ratio was about 1:30.

**Table 4:** Effect of breed, class of somatic cell counts, farm on cortisol concentration in milk and plasma (ln of pg/ml) of dairy cows in study 1. The model includes the linear effect of days in milking (DIM) and milk yield (kg/d)

	Milk			Plasma
	Mean	Se	Mean	Se
Breed				
IH	5.77	0.06^a^	7.81	0.11^Ns^
IS	5.62	0.07^b^	8.02	0.08^Ns^
Class				
#1	5.72	0.05^Ns^	7.84	0.09^Ns^
#2	5.55	0.15^Ns^	8.01	0.16^Ns^
#3	5.70	0.05^Ns^	7.90	0.07^Ns^
Farm				
FA	5.83	0.08^A^	8.24	0.10^A^
FB	5.44	0.09^B^	7.84	0.11^B^
FC	5.61	0.11^B^	7.27	0.12^C^
FD	5.92	0.06^A^	8.31	0.13^A^
DIM	−0.002	0.001^Ns^	−0.001	0.002^Ns^
Milk yield	0.010	0.007^Ns^	−0.006	0.011^Ns^
General Mean	5.72	0.06	7.98	0.09

*IH* Italian Holstein, *IS* Italian Simmental. *FA to FD* farm A to farm D, *se* standard error. *DIM* days in milking. Covariates appearing in the model are evaluated at the following values for Milk and Plasma cortisol of experiment 1: DIM = 142.25, Milk, kg/d = 33.61

^a^, ^b^ on the same column denote significant differences for *P* < 0.05; ^A^, ^B^, ^C^ on the same column denote significant differences for *P* < 0.01; ^Ns^: not significant

The effects of breed, class of SCC and farm on milk cortisol levels in study 2 are reported on Table 5. For IH cows, significantly higher values of milk cortisol (*P* < 0.01) in comparison to IS cows was observed. A significant effect of class of SCC was observed and in particular cows belonging to class 3 (i.e. SCC higher than 400.000/ml) showed the highest mean values of milk cortisol (*P* < 0.05). The farm effect was significant also in the study 2 (Table 5, *P* < 0.01), confirming the results obtained in the first study.

**Table 5:** Effect of breed, class of somatic cell counts and farm on cortisol concentration in milk of dairy cows in study 2. Data are reported as estimated means of natural logarithm of cortisol (pg/ml)

	Mean	Se
Breed		
IH	6.43	0.07^A^
IS	5.94	0.06^B^
Class		
#1	6.15	0.02^a^
#2	6.14	0.05^a^
#3	6.27	0.04^b^
Farm		
F1	6.02	0.05^AB^
F4	6.10	0.05^B^
F5	6.19	0.06^BC^
F10	6.05	0.07^AB^
F2	6.04	0.05^AB^
F3	6.33	0.06^C^
F6	5.85	0.08^A^
F7	6.37	0.04^C^
F8	6.36	0.05^C^
F9	6.01	0.06^AB^
DIM	0.001	0.001^Ns^
Milk yield	0.001	0.003^Ns^
General mean	6.19	0.03

Covariates appearing in the model are evaluated at the following values for Milk cortisol of Model 1: DIM = 159.57, Milk, kg/d = 31.30; *F1 to F10* farm 1 to farm10, *se* standard error, *DIM* days in milking

^a^, ^b^ on the same column denote significant differences for *P* < 0.05; ^A^, ^B^, ^C^ on the same column denote significant differences for *P* < 0.01; ^Ns^: not significant

## Discussion

The aim of the first study was to compare milk and blood cortisol concentrations and to consider its variability in milk in relation to farm, milk yield and DIM. In the model, also 3 classes of SCC and breed were included, even though the results have to be considered with caution since only 20 % of cows were sampled. The classification criteria for an inflammatory response using SCC were more than 200,000 SCC/ml, a threshold for inflammatory response indicating that a subclinical mastitis is occurring [18], and more than 400,000 SCC/ml, the upper limit indicated by the European Union for human consumption [19].

The overall mean milk cortisol concentration in study 1 was 330 pg/mL for the untransformed values (298 pg/mL for the exponential of log transformed values) and lies within the range of values reported by Verkerk et al. [20] and Fukasawa & Tsukada [11]. Also the concentration of serum cortisol were within the ranges reported by Gabai et al. [16]. Milk and blood cortisol concentrations were not correlated (Fig. 1), probably reflecting the episodic secretion of cortisol in blood sampled from restrained animals. These result do not agree with those of Gygax et al. [21], who reported that measurements of cortisol concentrations in milk and blood correlate closely. Also Shutt & Fell [22] and Verkerk et al. [20] reported a correlation between serum and milk cortisol, but the data obtained by these authors referred to free fraction of blood and milk cortisol [22] and were obtained from animals sampled after severe adrenal stimulation. Moreover, Verkerk et al. [20] suggested that milk cortisol can reflect serum concentration only within 2–4 h after the response to acute stressors of lactating cows. Similarly, Romero et al. [23] in goats reported a delay of 1.5 h between blood and milk cortisol increase in response to an acute stress. According to Fox et al. [24], adrenal cortisol secretion is transferred from blood to milk rapidly (within 4 h), but in the absence of sustained activation of HPA axis the transfer rate declines and cortisol is diluted later, as a function of milk yield and milking interval. Considering that in our trial cows are milked about every 12 h, cortisol concentration in milk is likely to represent a picture of the average blood cortisol variations in the previous 10 to 14 h window. It must also be considered that in our study the collection of blood was subsequent to milk sampling, the first referring to the acute secretion and the latter to the previous 12 h secretion. Moreover, as cortisol diffuses across the blood-milk barrier, a leak back from milk to plasma of the hormone can also be taken into account. Local regulation of cortisol production in mammary gland due to the activity of 11ß-hydroxysteroid dehydrogenase can be another factor interfering on the concentration of hormone in milk. However, these aspects are beyond the scope of the study that aimed not only to investigate the correlation between sampling sites but also to understand if the breed and farm effects were similar using the two biological fluids. As it can be seen from Table 4, the statistical effect of breed or farm factors differed from plasma to milk cortisol contents.

The difference of cortisol content in milk observed between breeds in the first study (Table 4) can be ascribed to the different ability to cope with milk yield for IH and IS. High yielding cows (IH) are selected for milk production whilst IS cows are dual purpose animals (www.anafi.it; www.anapri.it), (i.e. milk and meat production). The higher values of milk production observed in the present study for IH cows (Table 4) can arise from the catabolic activity of glucocorticoids [25]. Higher serum cortisol in Holstein in comparison the Montbeliarde-sired crossbred cows were reported by Mendonça et al. [26] during the transition period. Also Negrao & Marnet [27] found that Holstein cows with higher milk yield had higher levels of plasma cortisol. In this experiment, the authors sampled the cows 7 consecutive days using a catheter implanted into the jugular vein 72 h before the first sampling to avoid acute cortisol secretion linked to animal handling.

However, the more productive IH cows of FD showed the highest milk cortisol, but also the least productive IS cows of FA had significant higher milk cortisol than cows of FB and FC. It is well known that individual differences in response to stress are affected by both genetic [28] and environmental factors [29]. The observed differences of milk cortisol concentration between farms suggest that different rearing conditions, such as stocking rate, shape and type of cubicles and number of animals per productive group may affect the HPA axis activity, thus beholding the hypothesis that the effects of environment can superimpose the genetic background [30].

The effects of breed, class of SCC and farm on milk cortisol levels in the second study was investigated in a larger number of cows and farms (Table 5), with the aim to validate the results obtained in the pilot study. The effects of breed and farm on milk cortisol concentration were significant, as was evidenced in study 1, but not all the IH farms had higher milk cortisol than IS farms, suggesting that the interaction between genotype and environment on the HPA axis regulation deserves further investigation.

However, a unitary integrated complex consisting of a “psycho-sensitive stimuli/behavioural response” and “antigenic stimuli/immune response” is involved in the adaptation of the host and in the activation of the HPA axis [7]. Immune response that activates the production of regulatory cytokines stimulates the release of circulating glucocorticoids from the pituitary-adrenal axis Charmandari et al. [31]. The SCC in milk is considered an index of mammary gland inflammation and when SCC in milk exceed 200,000 cells/ml, an inflammatory response has been elicited (i.e. subclinical mastitis) [18]. In the present study a significant effect of SCC class on milk cortisol (*P* < 0.05) was calculated only for class 3 (SCC higher than 400,000 cells/ml), suggesting that an enhancement of cortisol release after HPA activation subsequent to an “antigenic stimuli/immune response” can be detected only when the inflammatory response is more severe. To better ascertain the use of milk cortisol as a biomarker of mammary gland health, its relationship with larger plethora markers of inflammation may be required. Previous studies conducted both on cows and goats did not find correlation between milk cortisol concentration and the level of SCC [32, 33]. However, according to Mehdid et al. [34] and Diaz et al. [32], if severe stress occurs, the concentration of cortisol in blood increases and SCC increases.

## Conclusions

Milk sampling can be performed directly in milking parlour without animal handling and overcomes some of the problems associated with other sampling sites, as blood, urine and faeces. For this reason, milk can be considered a preferential site of sampling in dairy cows to point out short term stimulation of the HPA axis. Moreover, it is likely that a time course analysis of milk cortisol can also indicate chronic conditions of animals.

The results of the study, obtained from a consistent number of lactating cows, suggest that milk cortisol concentration provides complementary information on the multifaceted activation of HPA axis. Considering that breed is a factor affecting milk cortisol concentration, a comparison between farms within the same breed can provide additional information about the wellbeing of the dairy cows. The relationship between milk cortisol and SCC is intriguing and could indicate that modifications of immune response can be detected using this marker at individual level within a herd.

However, these original results deserve further studies to investigate the physiological basis of the relationship between milk cortisol content and breed, environmental factors or healthy status of the animals.

## Abbreviations

HPA: Hypothalamic-pituitary-adrenal
SCC: Somatic cell count
IS: Italian Simmental
IH: Italian Holstein
FA: Farm A
FB: Farm B
FC: Farm C
FD: Farm D
DIM: Days in milking
F1: Farm 1
F2: Farm 2
F3: Farm 3
F4: Farm 4
F5: Farm 5
F6: Farm 6
F7: Farm 7
F8: Farm 8
F9: Farm 9
F10: Farm 10
TMR: Total mixed ration
BCS: Body condition score

## References

[1] Oltenacu PA, Broom DM (2010). The impact of genetic selection for increased milk yield on the welfare of dairy cows. Anim Welfare.

[2] Pritchard T, Coffey M, Mrode R, Wall E (2013). Genetic parameters for production, health, fertility and longevity traits in dairy cows. Animal.

[3] Banos G, Wall E, Coffey MP, Bagnall A, Gillespie S, Russell GC, McNeilly TN (2013). Identification of Immune Traits Correlated with Dairy Cow Health, Reproduction and Productivity. PLOSone.

[4] Sandri M (2014). Relation of rumen microbiome and blood transcriptome with the genetic merit in Italian Simmental and Italian Holstein cows.

[5] Nater UM, Whistler T, Lonergan W, Mletzko T, Vernon SD, Heim C (2009). Impact of acute psychosocial stress on peripheral blood gene expression pathways in healthy men. Biol Psychol..

[6] Sgorlon S, Colitti M, Asquini E, Ferrarini A, Pallavicini A, Stefanon B (2012). Administration of botanicals with the diet regulates gene expression in peripheral blood cells of Sarda sheep during ACTH challenge. Domes Anim Endocrinol..

[7] Amadori M, Stefanon B, Sgorlon S, Farinacci M (2009). Immune system response to stress factors. It J Anim Sci.

[8] Tsigos C, Chrousos GP (2002). Hypothalamic–pituitary–adrenal axis, neuroendocrine factors and stress. J Psychosom Res..

[9] Bertulat S, Fischer-Tenhagen C, Suthar V, Möstl E, Isaka N, Heuwieser W (2013). Measurement of fecal glucocorticoid metabolites and evaluation of udder characteristics to estimate stress after sudden dry-off in dairy cows with different milk yields. J Dairy Sci..

[10] Horst RL, Jorgensen NA (1982). Elevated plasma cortisol during induced and spontaneous hypocalcemia in ruminants. J Dairy Sci..

[11] Fukasawa M, Tsukada H (2010). Relationship between milk cortisol concentration and the behavioral characteristics of postpartum cows introduced to a new group. Animal Sci J..

[12] González-de-la-Vara MR, Valdez RA, Lemus-Ramirez V, Vázquez-Chagoyán JC, Villa-Godoy A, Romano MC (2011). Effects of adrenocorticotropic hormone challenge and age on hair cortisol concentrations in dairy cattle. Can J Vet Res.

[13] Jarrige R, INRA (1989). Ruminant Nutrition. Recommended Allowances and Feed Tables.

[14] Edmonson AJ, Lean IJ, Weaver LD, Farver T, Webster G (1989). 1989 A body condition scoring chart for Holstein dairy cows. J Dairy Sci..

[15] Waki T, Nakao T, Moriyoshi M, Kawata K (1987). A practical test of adrenocortical function in dairy cows: cortisol levels in defatted milk and its response to ACTH. J Coll Dairying..

[16] Gabai G, Mollo A, Marinelli L, Badan M, Bono G (2006). Endocrine and ovarian responses to prolonged adrenal stimulation at the time of induced CL regression. Reprod Domest Anim..

[17] SPSS (1997). Statistical Package for Social Science, Advanced Statistics 7.5.

[18] Hillerton JE (1999). Redefining mastitis based on somatic cell count. Bullett Internat Dairy Fed.

[19] Europa 2009 Regulation (EC) No. 853/2004 of the European Parliament and of the Council of 29 April 2004 laying down specific hygiene rules for food of animal origin (OJ L 226, 25.6.2004, p. 22) (with successive amendments and corrections consolidated).

[20] Verkerk GA, Phipps AM, Carragher JF, Mattews LR, Stelwagen K (1998). Characterisation of milk cortisol concentrations as a measure of short-term stress responses in lactating dairy cows. Anim Welfare.

[21] Gygax L, Neuffer I, Kaufmann C, Hauser R, Wechsler B (2006). Milk cortisol concentration in automatic milking systems compared with auto-tandem milking parlors. J Dairy Sci..

[22] Shutt DA, Fell R (1985). Comparison of total and free cortisol in bovine serum and milk or colostrum. J Dairy Sci..

[23] Romero G, Restrepo I, Muelas R, Bueso-Ródenas J, Roca A, Díaz JR (2014). Within-day variation and effect of acute stress on plasma and milk cortisol in lactating goats. J Dairy Sci..

[24] Fox L, Butler WR, Everett RW, Natzke RP (1981). Effect of adrenocortisotropin on milk and plasma cortisol and prolactin concentrations. J Dairy Sci.

[25] Elsasser TH, Klasing KC, Filipov N, Thompson F, Moberg GP, Mench JA (2000). The metabolic consequences of stress: targets for stress and priorities of nutrient use. The biology of animal stress.

[26] Mendonça LGD, Litherland NB, Lucy MC, Keisler DH, Ballou MA, Hansen LB, Chebel RC (2013). Comparison of innate immune response and somatotropic axis of Holstein and Montbéliarde-sired crossbred dairy cows during the transition period. J Dairy Sci.

[27] Negrao JA, Marnet PG (2006). Milk yield, residual milk, oxytocin and cortisol release during machine milking in Gir, Gir x Holstein and Holstein cows. Reprod Nutr Dev..

[28] Burrow HM (1997). Measurements of temperament and their relationship with performance traits of beef cattle. Anim. Breed Abstr..

[29] Kosako T, Imura T (1999). Effect of housing conditions and human contact on temperament of Japanese black calves. Anim Sci J..

[30] Gillespie CF, Phifer J, Bradley B, Ressler KJ (2009). Risk and Resilience: Genetic and Environmental Influences on Development of the Stress Response. Depress Anxiety..

[31] Charmandari E, Tsigos C, Chrousos G (2005). Endocrinology of the stress response. Annu Rev Physiol..

[32] Diaz JR, Alejandro M, Romero G, Moya F, Peris C (2013). Variation in milk cortisol during lactation in Murciano-Granadina goats. J Dairy Sci..

[33] Fukasawa M, Tsukada H, Kosako T, Yamada A (2008). Effect of lactation stage, season and parity on milk cortisol concentration in Holstein cows. Livest Sci..

[34] Mehdid MA (2009). Efecto del celo y del estrés sobre el recuento de Células Somaticas en la leche de la cabra [Effect of estrus and stress on somatic cell count in goat milk].

